# Physics-Guided CNN Detection of Crack-Associated Events from Embedded Fiber Bragg Grating Sensors

**DOI:** 10.3390/s26144556

**Published:** 2026-07-17

**Authors:** Yagiz Uğurveren, Alexander Gros, Enes Nohutcuoğlu, Tarik Tekoğlu, Kivilcim Yüksel, Karima Chah, Christophe Caucheteur

**Affiliations:** 1 Electromagnetism and Telecommunication Department, University of Mons, 7000 Mons, Belgium; aliyagiz.ugurveren@umons.ac.be (Y.U.); alexander.gros@umons.ac.be (A.G.); karima.chah@umons.ac.be (K.C.); 2 Department of Electrical Engineering, Izmir Institute of Technology, Urla 35430, Türkiye; kivilcimyuksel@iyte.edu.tr; 3 WEL Research Institute, Avenue Pasteur 6, 1300 Wavre, Belgium

**Keywords:** fiber Bragg grating, structural health monitoring, crack-event detection, convolutional neural network, Euler–Bernoulli beam theory, embedded sensors

## Abstract

Crack detection in composite structures remains a central challenge in structural health monitoring, particularly when sensing must rely on a small number of embedded multiplexed fiber Bragg gratings (FBGs). Here, we present a physics-guided convolutional neural network (CNN) framework for crack-associated event detection from multiplexed FBG interrogator signals acquired during the three-point bending of glass-fiber-reinforced polymer (GFRP) beams. The dataset was constructed from raw interrogator recordings and synchronized force–displacement metadata while preserving the cracked and non-cracked loading stages present in the experiments. Each candidate response was encoded by 13 synchronized optical, loading, and mechanics-guided descriptors, including Euler–Bernoulli expected strain and residual terms, where the residual denotes the difference between the measured response and the elastic response predicted by beam theory. A compact one-dimensional CNN operating on 30-response sequences was evaluated on 64 experimental runs under strict leave-one-run-out validation. At the selected operating point, the model reached window-level precision of 0.900, recall of 0.910, F1 score of 0.905, and balanced accuracy of 0.942, while the corresponding run-level decision reached a precision of 0.833, a recall of 1.000, an F1 score of 0.909, and a balanced accuracy of 0.969. Bootstrap resampling over runs yielded 95% confidence intervals of 0.787–0.978 for window-level F1 and 0.769–1.000 for run-level F1. To probe generalization beyond the initial fabrication batch, the final frozen pipeline was also tested once on seven later-batch runs from two newly manufactured specimens, where it reached a window-level precision of 0.908, a recall of 1.000, an F1 score of 0.952, a balanced accuracy of 0.969, an ROC-AUC of 0.979, a PR-AUC of 0.955, and perfect run-level classification. These results show that a compact sequence CNN, enriched with mechanics-guided strain interpretation, can extract robust crack-event signatures from multiplexed FBG measurements while preserving a simple and reproducible modeling pipeline.

## 1. Introduction

Crack detection remains challenging in structural health monitoring (SHM) because damage initiates locally, while maintenance decisions are made at the structural scale. Optical fiber sensing, and in particular fiber Bragg grating (FBG) sensing, is used for this purpose because of low weight, immunity to electromagnetic interference, embeddability, and multiplexing capability along a single fiber line [1,2,3]. These properties have led to the use of FBG strain sensing in civil, aerospace, and composite structures. In composite materials, previous studies have discussed both the use of embedded FBG networks and the challenges associated with sensor integration, protection, and reliability over time [4,5]. Despite progress in strain measurement, translating interrogator data into robust damage-state information at specimen level remains an open problem.

Recent work has explored learning approaches to interpret optical-fiber measurements. In FBG systems, machine learning has been used to track crack initiation and growth in metallic structures, combine long-gauge measurements with deep learning for damage identification, and process dynamic responses from FBG arrays using dimensionality reduction and convolutional neural networks (CNNs) [6,7,8]. Other studies have relied on multispectral feature fusion for fatigue crack monitoring or combined FBG sensing with deep learning for damage identification in composite materials [9,10]. Recent Sensors reviews further show that deep learning now occupies a central place both in SHM workflows and in optical-fiber-sensor data interpretation more broadly [11,12]. At the sensor-processing level, deep networks have also been applied directly to dense FBG spectra rather than only to post-processed scalar descriptors [13]. In parallel, machine learning has been employed to disentangle strain and temperature effects in multiplexed FBG arrays [14]. While these approaches show that learning algorithms can extract information on damage, they typically rely on engineered features, reduced representations, or specimen labels rather than addressing localized crack-event screening from reconstructed multiplexed interrogator recordings under run-grouped evaluation. Related developments beyond optical-fiber sensing also report CNN-based crack screening, SHM data-reconstruction pipelines, and acoustic-emission-based damage monitoring, which further motivate compact but well-validated detection baselines in this area [15,16,17,18,19,20,21].

More advanced damage-localization strategies based on deep learning have been reported primarily for distributed fiber optic sensing (DFOS). These include the deep learning-assisted monitoring of spatially distributed cracks and automated crack quantification from distributed strain measurements while accounting for nonlinear effects [22,23]. Related developments in SHM include CNN-based anomaly screening for monitoring data, attention-based neural networks for structural crack detection, neural network-based crack detection using strain gauges, hybrid approaches to damage localization, and physics-informed frameworks driven by data [24,25,26,27,28]. Although these studies show progress in crack interpretation from sensing data, they generally operate on distributed strain fields, dense sensor networks, image representations, or other sensing modalities. Multiplexed FBG interrogator data instead provide a small number of synchronized channels and a more indirect view of damage. This study therefore focuses on crack-event detection from multiplexed FBG measurements.

This study addresses that gap by analyzing short fixed-length interrogator windows extracted from the multiplexed interrogator output around repeated loading responses. The task is to identify crack-associated windows from the target FBG response within a run-level evaluation protocol that reflects specimen correlation. This problem can be informed directly by structural mechanics. Under Euler–Bernoulli beam theory, the surface strain in a slender beam subjected to bending is related to the applied load and the section geometry [29]. Deviations between the measured FBG response and this expected elastic behavior provide a natural basis for mechanics-guided interpretation.

Building on this idea, we summarize each peak-centered response by a small set of optical and mechanical descriptors. The first group describes the measured wavelength itself, for example, its median value and within-peak spread. The second group describes how far the current response has moved away from the early reference state of the same run; in practical terms, this is obtained by subtracting the median wavelength of the first loading group of that run. The third group contains the synchronized loading variables, namely, force, displacement, and air pressure. The final group contains mechanics-guided quantities derived from Euler–Bernoulli theory: the strain expected from the applied load, the strain inferred from the measured wavelength shift, and their residual, that is, the difference between the measured response and the elastic response predicted by beam theory. A compact temporal CNN then analyzes short sequences of such responses, and a run-level decision is obtained by combining the window probabilities within each experiment. This formulation keeps the learning architecture simple while turning limited multiplexed FBG measurements into a structured and physically interpretable detection problem.

The main contributions of the present work are threefold. First, we formulate crack-event detection from short multiplexed FBG interrogator windows under strict leave-one-run-out validation. Second, we show that Euler–Bernoulli-guided strain interpretation can be integrated directly into a compact CNN through synchronized mechanics-aware descriptors rather than through a heavy multi-branch architecture. Third, we show that this simple physics-guided pipeline preserves high-sensitivity specimen-level screening on the grouped internal evaluation and transfers to a small later-batch holdout from two newly manufactured specimens while also outperforming a logistic baseline in window-level discrimination.

## 2. Materials and Methods

### 2.1. FBG Inscription and Interrogation

Uniform FBGs were inscribed in single-mode optical fibers by the phase-mask technique using a NORIA FBG inscription system (NorthLab Photonics, Nacka, Sweden) equipped with a 193 nm excimer laser [30]. During inscription, the reflected spectra were monitored in real time with an optical spectrum analyzer. Fibers of approximately 30 cm and 60 cm were prepared according to the specimen dimensions, and sensing fibers containing single, dual, or triple gratings were fabricated for the initial experimental campaign. Each grating had a nominal length of 4 mm. For fibers with multiple FBGs, adjacent inscription sections were separated by approximately 1.5 cm. Three phase-mask wavelength bands were used so that the reflected peaks were distributed over separate spectral intervals around 1540–1545 nm, 1555–1560 nm, and 1570–1575 nm. Standard and hydrogen-loaded fibers were both tested during development, with the latter providing higher reflectivity. The inscription process was adjusted to obtain similar reflection levels across gratings so that the multiplexed interrogator signals maintained comparable signal-to-noise ratios.

### 2.2. Composite Fabrication and Sensor Embedding

**Figure 1:**
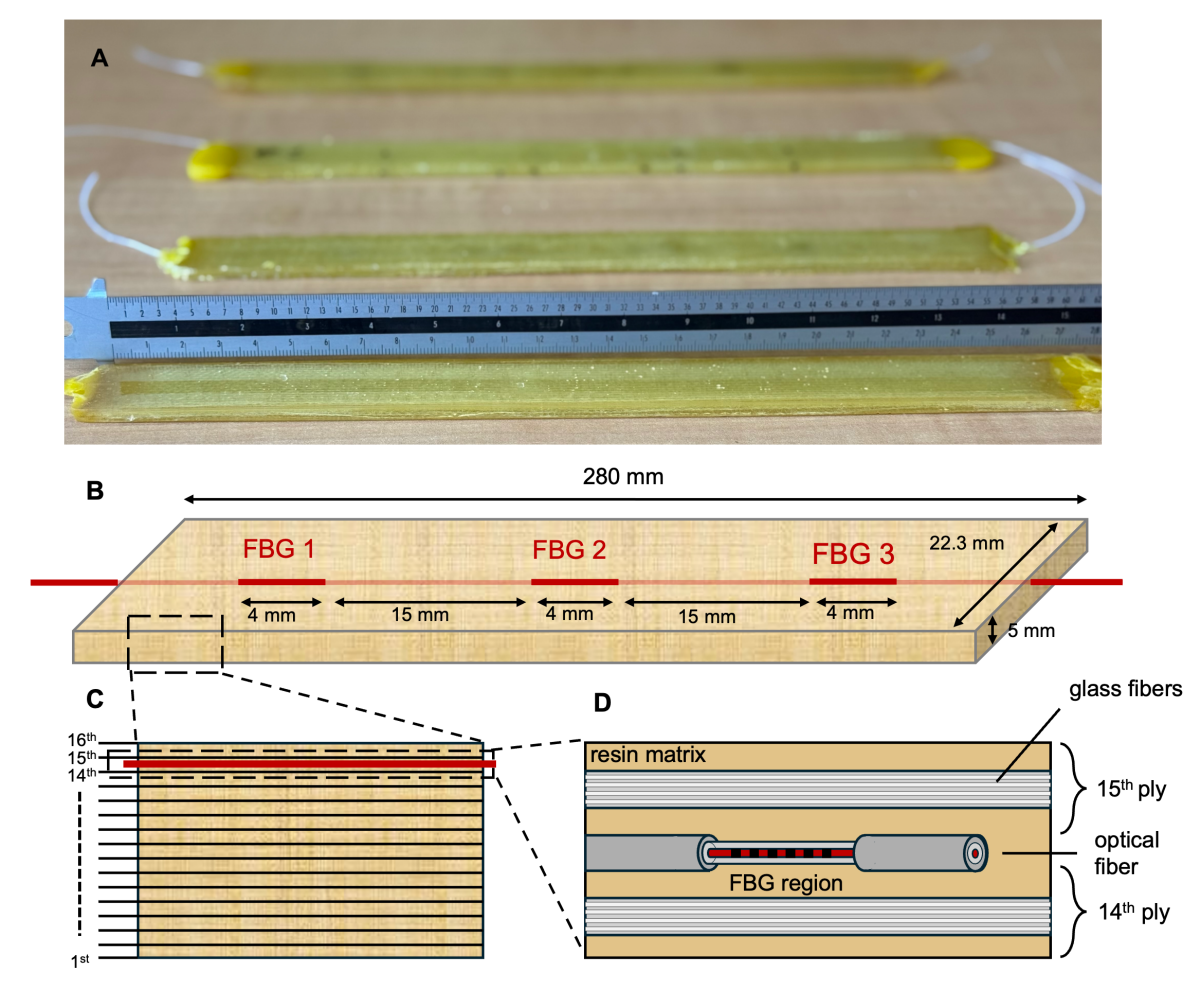
Fabricated composite specimens, specimen geometry, laminate layup, and local sensor embedding. Panel (**A**) shows representative hand-laminated glass-fiber-reinforced polymer specimens used in the three-point bending experiments, with a ruler provided for scale. Panel (**B**) shows the specimen geometry, nominal dimensions, and the positions of the three embedded FBGs along the optical fiber. Panel (**C**) shows a cross-section schematic of the 16-ply laminate, highlighting fiber placement between the 14th and 15th plies near the upper surface. Panel (**D**) shows a close view of this interply region, indicating the glass-fiber layers, resin matrix, embedded optical fiber, and the FBG sensing region.

Glass-fiber-reinforced polymer (GFRP) specimens were fabricated by hand layup using glass-fiber textiles and epoxy resin. The 16-ply laminate configuration was selected for the experiments after preliminary trials on 12-ply specimens to assess the influence of specimen stiffness on the bending response. Hand layup with embedded FBGs is established for composite monitoring, but strain transfer and local stress concentrations remain sensitive to sensor position and laminate architecture [4,31,32]. After impregnation, the laminates were cured in a climate chamber at 40 °C for 14 h. The embedded optical fiber was placed close to the upper surface of the laminate, typically two plies below the top face, so that the FBGs experienced a larger tensile or compressive strain response during bending rather than remaining close to the neutral axis. For the nominal 16-ply configuration, this corresponded to placing the sensing fiber between the 14th and 15th plies. The 12-ply specimens were not used for the final analysis because the three-point bending machine reached its maximum displacement too early, which prevented the acquisition of smooth strain curves. The 16-ply configuration was therefore used for data collection and analysis. Representative composite specimens together with schematics of the specimen geometry, laminate layup, and local sensor embedding are shown in Figure 1.

### 2.3. Three-Point Bending Experiments

**Figure 2:**
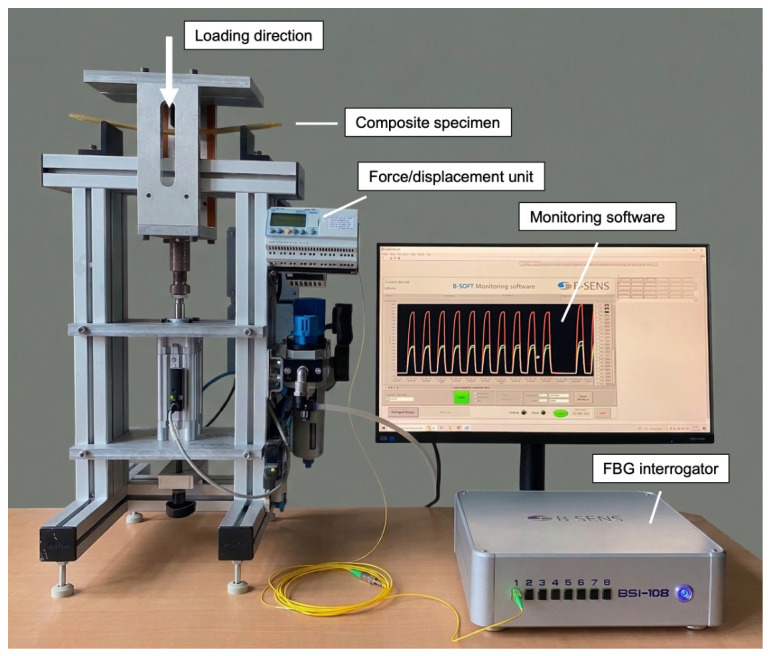
Three-point bending measurement chain and experimental setup. The image shows the instrumented three-point bending system used in the experiments, including the loading direction, the composite specimen, the force/displacement unit, the acquisition computer, and the BSI-108 FBG interrogator.

Mechanical testing was performed with a pneumatic three-point bending system instrumented to measure applied force and crosshead displacement. Each specimen was placed on two supports separated by a prescribed span, and load was applied at midspan. The actuator force was controlled through discrete air-pressure settings. For each selected pressure level, ten consecutive loading cycles were applied before moving to the next level. This protocol produced repeated measurements within a given loading block while progressively increasing the loading level on the specimen. During each test, the bending machine recorded force, displacement, and applied pressure, while the embedded FBGs were interrogated in parallel by the BSI-108 FBG interrogator from B-Sens (Mons, Belgium). The instrumented bending setup is shown in Figure 2. In the main grouped dataset, support spans of 110, 150, 190, 230, and 270 mm were investigated, and the nominal specimen width was 22.3 mm.

### 2.4. Elastic Calibration and Mechanics-Guided Loading Interpretation

The mechanics-guided part of the study is anchored in the linear elastic response of a slender beam under three-point bending. Similar FBG bending studies have shown that when embedding is well controlled, the measured wavelength shift can be interpreted consistently with beam theory strain fields in the elastic regime [5]. For a simply supported specimen loaded at midspan, the applied force generates a bending moment

(1) $$M=\frac{FL}{4},$$

where *F* is the applied force and *L* is the support span. According to Euler–Bernoulli beam theory, the longitudinal stress varies linearly with the distance *y* from the neutral axis [29],

(2) $$\sigma =\frac{My}{I},$$

where *I* is the second moment of area of the cross-section. For the rectangular specimens considered here,

(3) $$I=\frac{bh^{3}}{12},$$

with *b* the specimen width and *h* the specimen thickness. We assume linear elastic behavior before crack initiation, ε=σ/E, so the strain at the FBG position becomes

(4) $$\varepsilon =\frac{My_{\mathrm{FBG}}}{EI}=\frac{3FLy_{\mathrm{FBG}}}{Ebh^{3}},$$

where *E* is the effective Young’s modulus and $y_{\mathrm{FBG}}$ is the signed distance of the sensor from the neutral axis.

**Figure 3:**
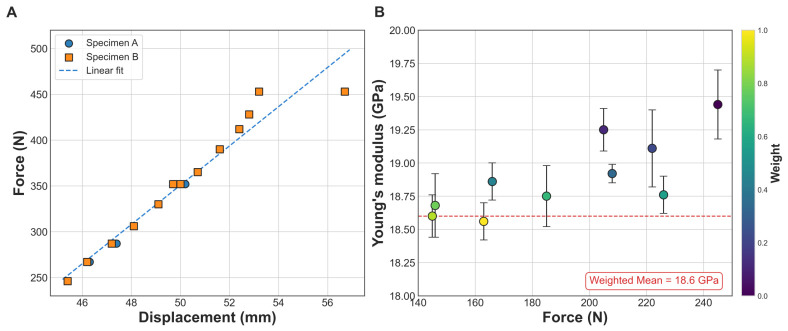
Calibration of the 16-ply specimens in the elastic regime. Panel (**A**) shows the measured force as a function of displacement for representative specimens, together with the fitted linear trend used to identify the initial elastic regime. Panel (**B**) shows the effective Young’s modulus estimated from calibration measurements before crack initiation using the FBG wavelength shifts and the Euler–Bernoulli bending relation. The colored markers indicate the relative weight assigned to each force-group estimate, based on the amount of calibration data available for that estimate, and the horizontal dashed line marks the resulting weighted mean of 18.6 GPa.

Before using loading-aware features in the learning pipeline, the elastic bending response was examined to verify that beam theory described the behavior before crack initiation. As shown in Figure 3A, the applied force increased approximately linearly with the measured displacement over the initial loading regime of the calibration specimens. The FBG strain sensitivity used to convert wavelength shifts into approximate axial strain was determined before the bending analysis using a calibrated Thorlabs optical-rail linear translation setup. In this calibration, the fiber was clamped between two stages, a known axial displacement was imposed over the measured gauge length, and the corresponding Bragg wavelength shift was recorded with the FBG interrogator. A linear fit of wavelength shift versus imposed strain gave the coefficient used here, 1.2 pm/μϵ. Using this calibrated coefficient, the measured wavelength shifts, and Equation (4), an effective Young’s modulus was estimated from pre-crack calibration data. Figure 3B shows the force-group modulus estimates together with a weighted mean, indicated by the horizontal dashed line. The weights differ across force groups according to the amount of calibration data available for each estimate, so groups supported by more data contribute more strongly to the final representative value. Despite these unequal contributions, the estimates remained tightly clustered, which supported the use of a single effective modulus of 18.6 GPa for mechanics-guided interpretation, a value consistent with reported elastic moduli for GFRP laminates and woven GFRP composites in the literature [33,34]. Across force groups, the estimated modulus values remained within 18.56–19.44 GPa, with a standard deviation of 0.275 GPa, which is why a single representative modulus was retained for the final mechanics-guided descriptors.

### 2.5. Data Acquisition, Peak Alignment, and Window Construction

The dataset was built from two synchronized sources: raw multiplexed interrogator recordings and experiment-level mechanical metadata. The FBG interrogator tracked the Bragg peak evolution of the active sensor channels over the 1510–1590 nm spectral range with sub-picometer wavelength resolution, while the bending setup recorded force, displacement, applied pressure, support span, and specimen geometry for each loading group. Crack-associated loading stages were annotated during the bending experiments using an auxiliary acoustic measurement chain. A microphone positioned near the specimen was connected to an oscilloscope, and the acoustic waveform was monitored in parallel with the FBG response during each loading cycle. Loading groups were labeled as crack-associated when an additional transient acoustic event, beyond the loading-start transient, was observed in the oscilloscope trace. The learning dataset was reconstructed directly from the raw interrogator text files and the associated experimental metadata rather than from pre-aggregated summary tables. This design preserves the waveform shape of each loading response, exposes the alignment path explicitly, and retains the information needed to test whether loading context improves detection.

Each experimental run was segmented into repeated loading responses centered on interrogator peaks. Two alignment strategies were examined: a fully automatic peak search performed directly on the raw interrogator traces, and a hybrid strategy using segment-boundary hints extracted from the aligned experimental records when those hints were available and internally consistent. The hybrid strategy produced the most stable run-level classification results and was therefore used for the main experiments. After segmentation, each selected response was resampled to a fixed-length representation while preserving the synchronized target channel and companion channels from the same multiplexed acquisition. This reconstruction step was central to the study because it converted raw interrogator files into repeatable learning units tied to the mechanical loading history.

The main grouped evaluation dataset contained 64 processed runs drawn from multiple physical specimens manufactured in the initial batch, rather than repeated measurements of a single specimen. After the final model configuration had been fixed, two newly manufactured follow-up specimens, one 12-layer beam and one 16-layer beam, provided seven additional runs that were reserved for later-batch holdout testing only. For most runs, 120 peak-centered responses were available, corresponding to 12 loading groups with 10 repetitions per group. Each response was associated with a run identifier, loading-group index, repetition index, sample indices in the original interrogator trace, its cracked or non-cracked state, and the synchronized loading metadata for the corresponding mechanical block. In addition to the raw interrogator channels, the dataset stored aligned loading traces for applied pressure, force, displacement, and an approximate elastic strain target derived from beam theory. This made it possible to derive compact sequence features from the optical response while preserving the synchronized mechanical context.

A compact split summary is given in Table 1. The main grouped evaluation comprised 64 processed runs, including 15 crack-positive and 49 crack-negative runs, while the later-batch holdout comprised 7 runs from the two follow-up specimens. The task is formulated as crack-event detection from multiplexed FBG measurements, with the cracked state being interpreted at the loading-group level and propagated to the sequence windows used for learning.

Mechanical metadata were aligned to the interrogator responses at the level of repeated loading blocks. Force, displacement, pressure, support span, specimen thickness, and specimen width were assigned to each selected response through the combination of run identifier and loading-group index. These synchronized quantities were then used to derive the loading features and the mechanics-guided residual terms. Because responses from the same run remained temporally and mechanically correlated, all evaluation protocols were performed at run level rather than by random splitting.

**Figure 4:**
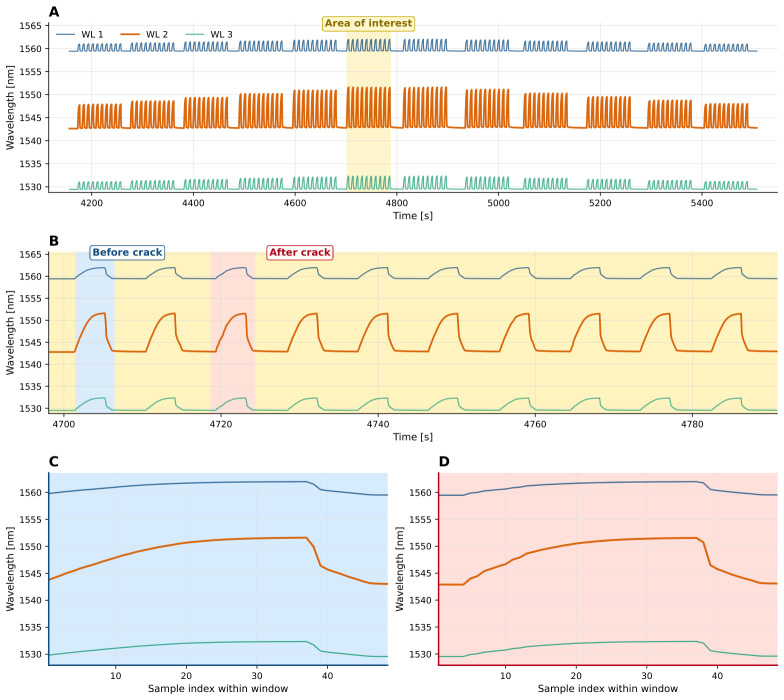
Example raw interrogator sequence and response hierarchy used for learning. Panel (**A**) shows the full multiplexed FBG interrogator run, with one representative loading group highlighted. Panel (**B**) shows the corresponding 10-response group on a shorter time scale, with responses before crack initiation and crack responses highlighted. Panels (**C**,**D**) show the individual responses before and after crack initiation, respectively, for the target FBG channel together with its two companion channels.

Figure 4 shows this hierarchy on one sequence. Figure 4A shows the full interrogator run together with one representative loading group. Figure 4B zooms into the corresponding 10-response group and highlights responses before crack initiation and crack responses. Figure 4C,D then show the individual responses used to illustrate the local waveform changes observed across the multiplexed FBG channels before and after crack initiation.

### 2.6. Sequence Representation and Selected Input Features

The final representation encodes each repeated peak-centered response as a compact feature vector instead of the full interrogator waveform. It was selected because it produced reproducible grouped detection results while keeping the role of the mechanics terms explicit. Accordingly, the network operates on a structured 30×13 derived-feature tensor reconstructed from the raw interrogator recordings, rather than on the raw waveform samples directly. Thirteen features were used in the final model: the median wavelength of the target channel, the within-response standard deviation of that wavelength, the target-channel shift relative to the early run baseline, synchronized force, synchronized crosshead displacement, applied air pressure, the change in that baseline-corrected wavelength shift across consecutive responses, the change in the displacement signal across consecutive responses, the Euler–Bernoulli expected elastic strain, the strain inferred from the measured wavelength sensitivity, a normalized difference between observed and expected strain, the change in that difference relative to the initial run baseline, and a binary flag indicating the reduced-size specimen subgroup identified in the source filenames. These features are summarized in Table 2.

Each learning sample comprised 30 consecutive responses from the same run, with stride 1. The label attached to each 30-response sequence was the maximum of the crack-event labels already present within that sequence and the label observed at a prediction horizon of 5 responses beyond the sequence end. This rule preserved sensitivity to transitions that emerged near the end of a local response history while keeping the sequence definition fixed across runs. Feature normalization was fitted on the training split only within each validation fold.

### 2.7. Selected Simple Sequence CNN

The classifier is a compact one-dimensional CNN operating on a 30×13 input array, where the 30 rows correspond to consecutive responses and the 13 columns correspond to the synchronized feature set described above. The network reads this array along the response order. In other words, it looks for short temporal patterns across consecutive loading responses rather than treating the 30 responses as independent points. The final classifier therefore analyzes sequentially organized derived descriptors rather than the full interrogator waveform itself.

The model uses three temporal convolution layers with 32, 64, and 128 channels, all with kernel size 12 and padding 6. Each convolution is followed by a rectified linear unit activation, which keeps positive activations and suppresses negative ones. Adaptive average pooling then summarizes the full 30-response sequence into a single 128-dimensional vector. This vector is passed to a classifier head composed of a 64-unit fully connected layer, another rectified linear unit activation, a dropout of 0.2, and a final linear output layer producing one crack-event logit. During training, this logit is optimized directly by weighted binary cross-entropy with logits. During inference, a sigmoid transformation converts it into a crack-event probability between 0 and 1.

**Figure 5:**
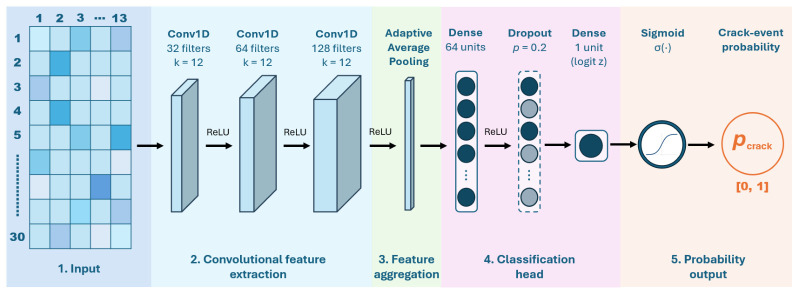
Compact one-dimensional sequence CNN used for the main result. Each input sample is a 30×13 feature tensor built from 30 consecutive peak-centered responses and 13 synchronized optical, loading, and mechanics-guided features. The network applies three temporal convolution layers with 32, 64, and 128 filters, all with kernel size 12, each followed by rectified linear unit activation, then adaptive average pooling, a 64-unit dense layer with rectified linear unit activation, dropout with probability 0.2, and a sigmoid transformation of the final logit to obtain crack-event probability.

This architecture was selected because it keeps the modeling pipeline simple while still allowing the network to learn short-range temporal motifs such as progressive drift, abrupt jumps, or repeated loading patterns across consecutive responses. Figure 5 shows the CNN used for the main result.

### 2.8. Training, Model Selection, and Evaluation

This study uses grouped evaluation because neighboring responses from the same run are highly correlated. The main protocol therefore uses leave-one-run-out validation, implemented as leave-one-group-out cross-validation with the run identifier as grouping variable. In this setting, each held-out fold corresponds to an unseen experimental run, the primary predictions are generated at the 30-response window level, and the run-level decision is obtained by combining the sequence predictions within that run. For the reported results, this run-level aggregation is a max-over-windows rule: a run is marked positive if at least one of its windows exceeds the selected probability threshold.

The selected operating point used the reconstructed aligned dataset described above. Training used weighted binary cross-entropy to account for class imbalance, a fixed random seed, a batch size of 128, Adam optimization with learning rate $10^{-3}$ and weight decay $10^{-4}$, a maximum of 8 epochs, and early stopping with patience 3 on the inner validation split. The final evaluation set yielded 5204 valid sequence windows, including 1045 positive windows under the fixed window target used in the main experiment. This configuration preserved a compact training procedure while providing stable grouped generalization across held-out experiments.

Within each outer leave-one-run-out fold, the decision threshold was tuned only on validation outputs coming from the training-side data of that fold, so the held-out test run was not used during threshold selection. The selected physics-guided operating point uses a fixed threshold of 0.45. No additional post-hoc physics filter was applied to the predictions reported here. Instead, the mechanics contribution is already embedded in the input tensor through the expected elastic strain and strain-residual descriptors derived from the synchronized loading history.

**Figure 6:**
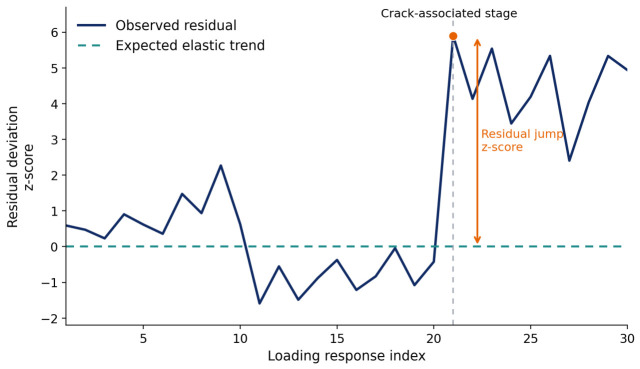
Representative load–wavelength residual trace from the selected dataset. The solid curve shows the signed normalized strain residual between observed and expected strain over a 30-response sequence, while the dashed horizontal reference indicates the elastic reference level. The highlighted jump at the crack-associated stage illustrates the mechanics-guided discrepancy signal from which the residual descriptors were derived.

Figure 6 illustrates this mechanics-guided signal on a representative crack-positive sequence from the selected dataset. The plotted deviation is computed from the difference between the observed strain inferred from the target FBG response and the Euler–Bernoulli elastic expectation under the synchronized loading history. The marked jump at the crack-associated stage is representative of the residual behavior summarized by the mechanics-aware descriptors used in the CNN input tensor.

Window-level precision, recall, $F_{1}$, and balanced accuracy are reported first because they reflect the local crack-event discrimination learned by the model under run-grouped validation. Run-level metrics are then reported as specimen-scale screening indicators derived from the same window predictions.

### 2.9. Statistical Reporting and Validation Scope

Because neighboring responses within the same experiment are not independent, all uncertainty estimates were computed by bootstrap resampling at run level. In addition to the point estimates reported for the main operating point, 95% confidence intervals were estimated by the nonparametric bootstrap resampling of the 64 evaluated runs with replacement over 5000 iterations. For window-level metrics, each resampled run contributed all of its corresponding windows; for run-level metrics, each resampled run contributed a single experiment-level label. This provides uncertainty bands tied to the independent experimental units rather than to the much larger number of overlapping response windows.

After the grouped model-development study had been completed, two additional specimens were manufactured and tested later under the same laboratory setup. Their seven runs, all acquired at 110 mm span, were not used for model fitting, epoch selection, or threshold selection. Because the final architecture, feature set, and operating threshold were fixed before these later specimens were tested, this follow-up evaluation provides a limited specimen-level external validation. It does not replace broader multi-setup validation, but it is stronger than another resplit of the original batch. The present work therefore emphasizes grouped within-campaign validation, run-bootstrap confidence intervals, and explicit separation between local window-level discrimination and specimen-level run decisions.

## 3. Results

### 3.1. Window-Level and Run-Level Detection Performance

The main evaluation was performed with strict leave-one-run-out validation on the dataset. Table 3 reports the window-level metrics of the physics-guided CNN together with the corresponding run-level decision obtained from the same predictions.

Bootstrap resampling over the 64 evaluated runs showed that the main result remained stable under grouped uncertainty estimation. At window level, the selected operating point yielded an $F_{1}$ of 0.905 with a 95% bootstrap confidence interval of 0.787–0.978, while the balanced accuracy was 0.942 with a 95% interval of 0.871–0.989. After combining window predictions at run level, the $F_{1}$ was 0.909 with a 95% bootstrap confidence interval of 0.769–1.000, and the balanced accuracy was 0.969 with a 95% interval of 0.930–1.000. These intervals show that the reported performance remains strong when uncertainty is estimated over independent experiments rather than over overlapping windows.

### 3.2. Later-Batch Holdout Validation and Baseline Comparison

To probe whether the method transferred beyond the initial fabrication batch, the final frozen pipeline was evaluated once on seven later-batch runs from two newly manufactured specimens, four crack-positive and three crack-negative, all acquired at 110 mm span. At the window level, the physics-guided CNN reached a precision of 0.908, a recall of 1.000, an $F_{1}$ of 0.952, a balanced accuracy of 0.969, an ROC-AUC of 0.979, and a PR-AUC of 0.955, as shown in the Table 4 below. At the run level, it correctly detected all four positive runs and correctly rejected all three negative runs, yielding precision, recall, $F_{1}$, and balanced accuracy of 1.000.

**Figure 7:**
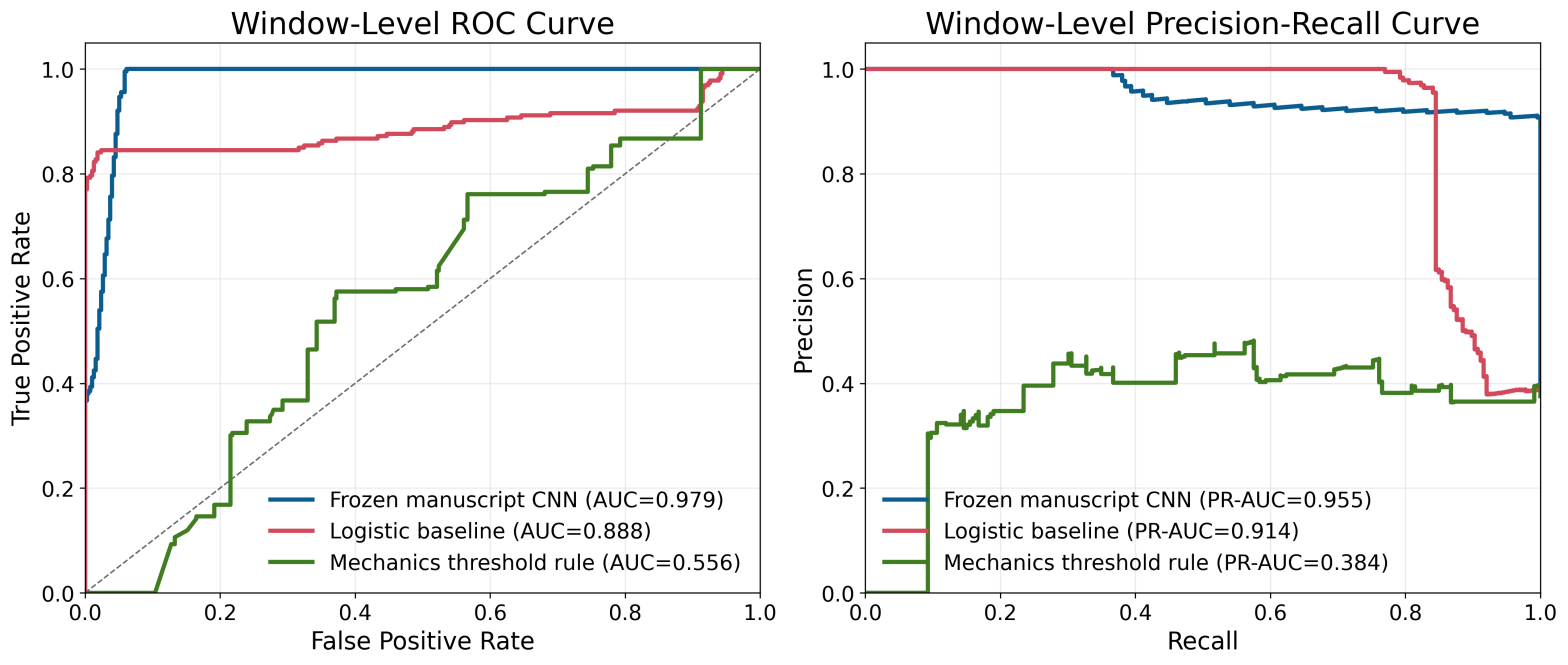
Receiver operating characteristic and precision–recall curves for the frozen physics-guided CNN, the logistic baseline, and the simple mechanics threshold rule on the later-batch holdout.

The simple mechanics threshold rule happened to flag all seven runs correctly at run level on this very small holdout, but its window-level discrimination was weak, with a window $F_{1}$ of 0.160, an ROC-AUC of 0.556, and a PR-AUC of 0.384, as depicted in the Figure 7 below. The logistic baseline was materially stronger than the threshold rule, yet it remained clearly below the CNN at the window level and also lost run-level precision. This comparison indicates that the contribution of the proposed pipeline is not merely any run-level alarm but a model that preserves specimen-level screening while ranking and localizing crack-associated windows more reliably than the simpler baselines.

### 3.3. Effect of Euler–Bernoulli Features

To isolate the effect of the mechanics-guided terms, we compared the selected 13-feature model with a matched 9-feature model that used the same grouped protocol but excluded the Euler–Bernoulli-derived descriptors. For each model, the probability threshold was tuned on the corresponding validation outputs. The comparison is reported in Table 5.

The optical-loading model without Euler–Bernoulli features already performed strongly at the window level. However, the addition of the mechanics-guided descriptors produced the stronger run-level screening point, increasing run-level recall from 0.933 to 1.000 and run-level $F_{1}$ from 0.875 to 0.909. In the present study, the principal benefit of the physics terms was therefore not a universal increase in every window-level metric but a more reliable experiment-level screening behavior with no missed crack-positive runs.

### 3.4. Confusion-Matrix View of the Selected Operating Point

**Figure 8:**
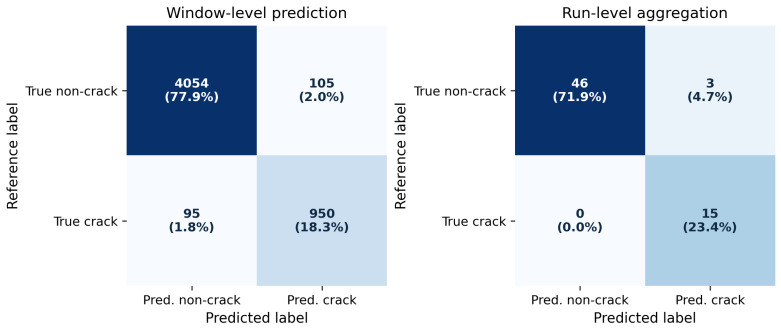
Confusion matrices for the selected physics-guided CNN operating point. The (**left**) panel reports the primary window-level predictions under strict leave-one-run-out validation. The (**right**) panel reports the corresponding run-level decision obtained by marking a run positive when at least one of its windows crosses the selected threshold.

The confusion matrices of the selected operating point are shown in Figure 8. At the window level, the model produced 4054 true negatives, 105 false positives, 95 false negatives, and 950 true positives. This distribution is consistent with the balanced precision and recall reported in Table 3, showing that the compact CNN separates crack-associated windows effectively while keeping both error types limited.

At the run level, combining the same window predictions yielded 46 true-negative runs, 3 false-positive runs, 0 false-negative runs, and 15 true-positive runs. This run-level decision preserved perfect sensitivity to crack-positive runs while limiting overprediction to three negative runs in the present campaign.

### 3.5. False-Positive Pattern Analysis

The confusion matrices summarize the overall error counts, but the false-positive behavior was not uniform across runs. Here, a loading group denotes one pressure-step block within a run, typically comprising 10 repeated loading cycles. Table 6 summarizes the three negative runs that were flagged positive at the run level in the grouped internal evaluation. Two of the three false-positive runs occurred at 230 mm span. The most persistent case was a 230 mm, 12-layer negative run in which positive windows extended from loading groups 6 to 11. By contrast, the 110 mm, 16-layer negative run and the other 230 mm, 12-layer negative run showed only isolated false-positive excursions around loading group 5.

Additional false-positive windows in crack-positive runs were limited to pre-onset segments of one 190 mm, 16-layer run, where the first labeled positive group was 7 but false positives already appeared in groups 5–6, and one 270 mm, 12-layer run, where a single false-positive window appeared in group 2 before the first labeled positive group 3. On the later-batch holdout, no negative run was falsely flagged at run level. The 23 window-level false positives on that split were confined to one cracked 16-layer follow-up run and appeared in groups 3 and 6–8, so they behaved as early over-alerting within a true positive run rather than as a healthy-run false alarm.

### 3.6. Mechanics-Guided Interpretation of the Detection Pipeline

The mechanics-guided contribution of the pipeline appears directly in the selected feature space. Euler–Bernoulli theory provides the elastic strain expectation used to relate applied force, specimen geometry, and the healthy loading response. The synchronized force and displacement variables anchor this expectation to the actual mechanical state of each loading block. The residual descriptors then quantify departures from the expected elastic behavior and expose precisely the kind of load–wavelength inconsistency that accompanies crack-associated events. This combination explains why a compact CNN can remain simple while still reaching strong local and run-level detection performance.

## 4. Discussion

These results show that crack-event detection is feasible from short peak-aligned responses of multiplexed FBG interrogator data when synchronized loading metadata and mechanics-guided residual descriptors are incorporated into the pipeline. The selected operating point reached balanced window-level detection while also preserving perfect run-level recall on the present dataset. This is important because it shows that useful specimen-level damage information can be extracted from compact multiplexed FBG measurements with a method that remains both interpretable and computationally light.

The later-batch holdout is important for interpreting possible overfitting. Because the two follow-up specimens were manufactured after model development and were never used for training, epoch selection, or threshold tuning, their successful evaluation weakens a pure memorization explanation.

The results also show that metric choice matters. Window-level scores capture the local discrimination learned by the model, whereas run-level scores capture the specimen-scale screening behavior after combining the window predictions of one experiment. Reporting both views is therefore useful in the present SHM setting: the first documents how well short loading histories are classified, and the second documents whether an unseen experimental run is flagged correctly.

The baseline comparison also helps position the contribution of the proposed method. A flattened logistic baseline lost substantial window-level precision and run-level precision on the later-batch holdout, while a simple mechanics threshold rule showed poor window-level ranking despite a perfect run-level outcome on this very small set. The contribution of the present framework is therefore the combination of compact sequential modeling, synchronized mechanics-aware descriptors, and reproducible grouped evaluation that yields both strong local-event discrimination and reliable specimen-level screening from sparse multiplexed FBG measurements.

Mechanics plays a practical role in the present study by structuring the detection pipeline around synchronized loading information. Force and displacement traces, expected elastic strain, and baseline-corrected load–wavelength features provide structural constraints that make the CNN sensitive to departures from healthy bending behavior rather than to wavelength variation alone. This supports the broader idea that structural priors can strengthen learning from sparse optical measurements when the sensing configuration does not provide rich spatial information.

The reduced-size specimen flag should also be interpreted cautiously. It was included as a subgroup-calibration covariate rather than as a crack proxy. When this covariate was removed and the threshold was re-tuned only on training-side validation outputs, later-batch run-level $F_{1}$ dropped from 1.000 to 0.800, with two of the three negative follow-up runs becoming false positives. This suggests that the flag helps calibrate systematic subgroup differences, but it does not by itself solve the crack-detection task.

The architecture is deliberately compact. Rather than relying on a heavy network, the present framework obtains its performance from the joint use of synchronized optical and mechanical descriptors, strict grouped evaluation, and a sequence model that is easy to reproduce. This simplicity is an advantage for reproducibility and makes the method straightforward to transfer to future experimental campaigns with richer sensor layouts or larger datasets.

The bootstrap confidence intervals sharpen the interpretation of the reported scores by quantifying uncertainty at the level of independent experiments rather than at the level of overlapping windows. They therefore complement the point estimates with a run-level view of stability, which is the relevant scale for the present screening task. Dedicated robustness studies for temperature drift, bonding variability, and broader cross-setup transfer remain necessary before stronger deployment claims can be made.

## 5. Conclusions

This study demonstrates that a compact CNN, combined with Euler–Bernoulli-guided strain interpretation, can detect crack-associated events effectively from multiplexed FBG interrogator measurements. On strict leave-one-run-out evaluation, the selected physics-guided operating point reached a window-level precision of 0.900, a recall of 0.910, an $F_{1}$ of 0.905, and a balanced accuracy of 0.942, while the corresponding run-level decision reached a precision of 0.833, a recall of 1.000, an $F_{1}$ of 0.909, and a balanced accuracy of 0.969. A later-batch holdout from two newly manufactured specimens further showed that the frozen final pipeline transferred beyond the original fabrication batch, reaching window-level ROC-AUC of 0.979 and PR-AUC of 0.955, with perfect run-level screening on seven follow-up runs. The mechanics-guided descriptors did not improve every window-level metric uniformly, but they strengthened the selected specimen-level screening behavior while preserving a simple, interpretable pipeline. These results show that synchronized loading information and simple mechanics-guided residual features can strengthen crack-event screening without requiring a heavy architecture.

Future work should now test the framework on independent fabrication campaigns and broader specimen geometries to assess generalization beyond the present within-campaign dataset. It would also be valuable to extend the approach to richer sensing layouts, including additional embedded FBG channels and more variable operating conditions, in order to evaluate the robustness of the mechanics-guided descriptors in less controlled settings. Finally, the present binary screening formulation could be expanded toward earlier crack-onset warning, sensor-level localization, and eventual damage-severity estimation, which would move the method closer to practical SHM deployment.

## Figures and Tables

**Table 1:** Compact summary of the evaluated grouped split and the later-batch holdout split.

Split	Source	Runs(pos./neg.)	Windows(pos./neg.)	Spans(mm)
Main grouped evaluation	Initial fabrication batch	64 (15/49)	5204 (1045/4159)	110:11, 150:20 190:16, 230:8 270:9
Later-batch holdout	Two follow-up specimens	7 (4/3)	602 (226/376)	110:7

**Table 2:** Input features used by the physics-guided sequence CNN.

Feature	Description
WL median wavelength	Median wavelength of the target FBG channel within the current peak-centered response.
WL wavelength spread	Standard deviation of the target-channel wavelength within the current response.
WL shift from run baseline	Median WL wavelength minus the median WL wavelength of the first loading group of the same run.
Force	Applied force assigned to the aligned loading block.
Displacement	Crosshead displacement assigned to the aligned loading block.
Air pressure	Pneumatic pressure assigned to the aligned loading block.
Change in WL baseline shift	Difference between the current and previous values of the WL shift from run baseline within the same run.
Change in displacement	Difference between the current and previous displacement values within the same run.
Expected elastic strain	Strain predicted from force and geometry using the Euler–Bernoulli bending relation.
Observed strain from wavelength sensitivity	Strain inferred from the target-channel wavelength shift using the fixed 1.2 pm/με strain-sensitivity calibration.
Normalized strain residual	Signed difference between observed and expected strain, divided by $\max(|\varepsilon_{\mathrm{expected}}|,10^{-6})$.
Change in strain residual from baseline	Current normalized strain residual minus the median normalized strain residual observed in the first loading group of the same run.
Reduced-size specimen flag	Binary subgroup covariate identifying the reduced-size specimen subgroup encoded in the source filenames; it is not a direct crack label.

**Table 3:** Main results for the selected physics-guided CNN operating point on the selected dataset.

Evaluation Level	Precision	Recall	$F_{1}$	Balanced Accuracy
Window level	0.900	0.910	0.905	0.942
Run level	0.833	1.000	0.909	0.969

**Table 4:** Later-batch holdout comparison between the frozen manuscript CNN and two simpler baselines tuned only on training-side validation outputs from the original batch.

Model	W. Prec.	W. Rec.	W. $F_{1}$	W.ROC-AUC	W.PR-AUC	R. $F_{1}$
Frozen physics-guided CNN	0.908	1.000	0.952	0.979	0.955	1.000
Logistic baseline	0.389	0.920	0.547	0.888	0.914	0.727
Mechanics threshold rule	0.324	0.106	0.160	0.556	0.384	1.000

**Table 5:** Effect of adding Euler–Bernoulli-derived features. Thresholds were tuned separately for each model on the grouped validation outputs.

Model	Level	Precision	Recall	$F_{1}$	Balanced Accuracy
Without Euler–Bernoulli features	Window	0.950	0.900	0.924	0.944
Without Euler–Bernoulli features	Run	0.824	0.933	0.875	0.936
With Euler–Bernoulli features	Window	0.900	0.909	0.905	0.942
With Euler–Bernoulli features	Run	0.833	1.000	0.909	0.969

**Table 6:** Run-level false-positive negative runs in the grouped internal evaluation.

Support Span (mm)	Layers	False-Positive Loading Groups
110	16	5 only
230	12	6–11
230	12	5 only

## Data Availability

The data supporting the reported results are available from the corresponding author upon reasonable request.

## Abbreviations

The following abbreviations are used in this manuscript:

CNN	Convolutional neural network
DFOS	Distributed fiber optic sensing
FBG	Fiber Bragg grating
GFRP	Glass-fiber-reinforced polymer
SHM	Structural health monitoring
WL	Wavelength
